# Identification and Characterisation of Nontuberculous Mycobacteria in African Buffaloes (*Syncerus caffer*), South Africa

**DOI:** 10.3390/microorganisms10091861

**Published:** 2022-09-17

**Authors:** Charlene Clarke, Tanya J. Kerr, Robin M. Warren, Léanie Kleynhans, Michele A. Miller, Wynand J. Goosen

**Affiliations:** DSI-NRF Centre of Excellence for Biomedical Tuberculosis Research, SAMRC for Tuberculosis Research, Division of Molecular Biology and Human Genetics, Faculty of Medicine and Health Sciences, Stellenbosch University, Tygerberg 7505, South Africa

**Keywords:** African buffaloes, bovine tuberculosis, *cfp*-10, *esat*-6, *hsp*65, nontuberculous mycobacteria, *rpoB*, *Syncerus caffer*

## Abstract

Diagnosis of bovine tuberculosis (bTB) may be confounded by immunological cross-reactivity to *Mycobacterium bovis* antigens when animals are sensitised by certain nontuberculous mycobacteria (NTMs). Therefore, this study aimed to investigate NTM species diversity in African buffalo (*Syncerus caffer*) respiratory secretions and tissue samples, using a combination of novel molecular tools. Oronasal swabs were collected opportunistically from 120 immobilised buffaloes in historically bTB-free herds. In addition, bronchoalveolar lavage fluid (BALF; *n* = 10) and tissue samples (*n* = 19) were obtained during post-mortem examination. Mycobacterial species were identified directly from oronasal swab samples using the Xpert MTB/RIF Ultra qPCR (14/120 positive) and GenoType CM*direct* (104/120 positive). In addition, all samples underwent mycobacterial culture, and PCRs targeting *hsp*65 and *rpoB* were performed. Overall, 55 NTM species were identified in 36 mycobacterial culture-positive swab samples with presence of *esat*-6 or *cfp*-10 detected in 20 of 36 isolates. The predominant species were *M. avium* complex and *M. komanii.* Nontuberculous mycobacteria were also isolated from 6 of 10 culture-positive BALF and 4 of 19 culture-positive tissue samples. Our findings demonstrate that there is a high diversity of NTMs present in buffaloes, and further investigation should determine their role in confounding bTB diagnosis in this species.

## 1. Introduction

The *Mycobacterium* genus consists of more than 170 species of mycobacteria, which includes the 11 members of the *Mycobacterium tuberculosis* complex (MTBC) and nontuberculous mycobacteria (NTM), also called mycobacteria other than tuberculosis (MOTT) [1,2]. The most important members of the MTBC are *M. tuberculosis* and *M. bovis,* which can cause severe disease in humans and animals, respectively [3]. However, with advances in molecular technology, the number of identified mycobacterial species is increasing, with species differing markedly in pathogenicity, drug response, virulence, environmental adaptation, and growth characteristics [2,3,4]. 

Nontuberculous mycobacteria are widely distributed in a variety of environments, such as soil and water, and although they have long been considered harmless environmental organisms, there is growing evidence of their importance in human and animal health [5]. Various NTM species are opportunistic pathogens and can cause disease in humans, as well as various livestock and wildlife species [3,5]. Collectively, diseases caused by NTMs are referred to as mycobacteriosis. In addition to the potential to cause disease, NTM infections in animals can interfere with bovine tuberculosis (bTB) diagnostic tests, since they can cause cross-reactive immune responses to antigens shard with MTBC; this has been observed when performing tuberculin skin tests and cytokine release assays [6,7]. 

Diagnosis of *M. bovis* infection in bovids, including African buffaloes (*Syncerus caffer*), which are important bTB wildlife maintenance hosts, typically relies on the early detection of cell-mediated immune (CMI) responses to *M. bovis* antigens, such as *M. bovis* purified protein derivative (bovine PPD) or mycobacterial peptides, early secretory antigen target 6 kDa (ESAT-6) and culture filtrate protein 10 kDa (CFP-10) [8,9,10]. Since a positive test result leads to culling and quarantine of the herd, false positive results due to cross-reactivity to NTMs can therefore lead to unnecessary losses of animals as well as income from sales, hunting and tourism [11].

In South Africa, movement of buffaloes requires screening for bTB [12]. The presence of environmental mycobacteria can confound interpretation of immunological tests, and it is therefore important to identify and characterise mycobacteria that are found in this species, to improve diagnostic accuracy. With advances in molecular techniques that can discriminate between mycobacterial species, detailed investigations of the role that NTMs play in sensitising animals can be performed. Therefore, this study aimed to identify and characterise NTM species diversity in respiratory secretions and tissue samples collected from African buffaloes, using a combination of available molecular tools (i.e., Xpert MTB/RIF Ultra qPCR assay, GenoType CM*direct* line-probe assay, PCRs, and Sanger sequencing). In addition, NTM species were screened for the presence of genes (*esat*-6 and *cfp*-10) that express antigens shared with *M. bovis,* as a foundation to understanding cross-reactive immune responses in buffaloes. 

## 2. Materials and Methods

### 2.1. Animals and Sample Collection

**Figure 1:**
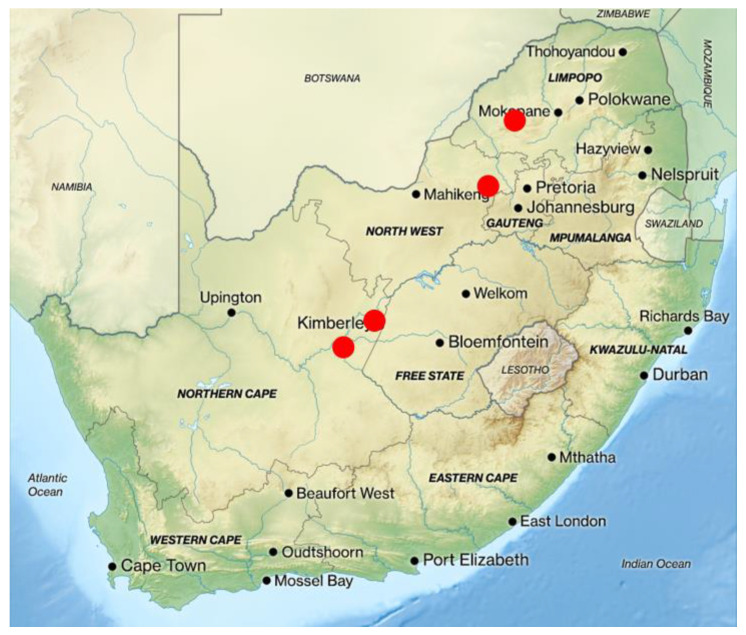
Map of South Africa indicating the sampling sites of buffaloes on private game reserves (red dots).

Oronasal swabs were opportunistically collected from African buffaloes (*n* = 120; cohort 1) at four historically *M. bovis* free game reserves in South Africa (Figure 1; North West, Limpopo and Northern Cape Provinces) between 2020 and 2021. Reserves were selected based on recent unexpected *M. bovis* positive test results from in vitro whole blood mycobacterial antigen cytokine release assays (CRA) or in vivo single comparative intradermal tuberculin tests (SCITT). Positive test results were suspected to be in response to NTM infection since *M. bovis* has not previously been isolated from post-mortem samples from any of these herds. Animals selected for this study included some with positive CRA results.

**Figure 2:**
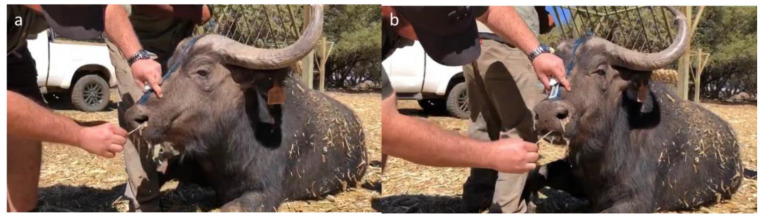
Collection of oronasal secretions from immobilised African buffaloes using swabs. (**a**) Swab collection of nasal secretions; (**b**) Swab collection from the mouth using the same swab as in (**a**).

**Figure 3:**
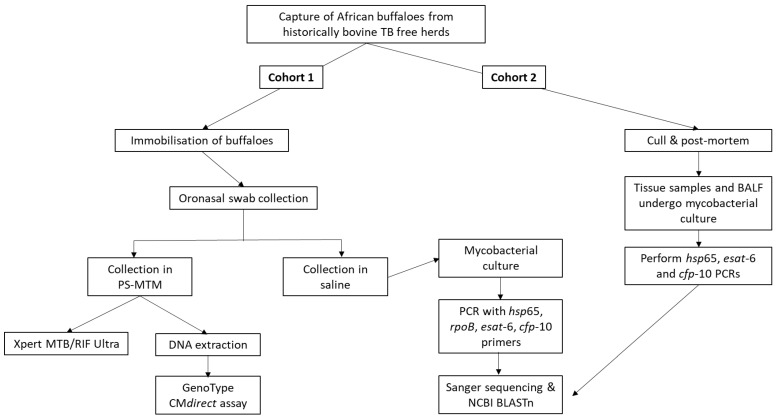
Flow chart indicating the study methods involved in the detection and characterisation of nontuberculous mycobacteria in African buffaloes. PS-MTM, PrimeStore^®^ Molecular Transport Medium; BALF, bronchoalveolar lavage fluid.

Following immobilisation of buffaloes for routine bTB testing procedures, as previously described [13], two swabs per animal were opportunistically collected from the oronasal secretions (Figure 2). One swab was stored at room temperature in PrimeStore^®^ Molecular Transport Medium (PS-MTM, 5 mL, Longhorn Vaccines & Diagnostics LLC, Bethesda, MD, USA), and the other swab (FLOQswab, Copan Diagnostics, Murrieta, California, USA) in saline (1.5 mL) at 4 °C until further testing (Figure 3), as previously described [14]. 

A second cohort (cohort 2) of buffaloes from the same herds as cohort 1, did not have oronasal swab samples collected, but were culled (based on their suspect bTB test results) and had tissues (*n* = 19) and bronchoalveolar lavage fluid (BALF; *n* = 10) collected for mycobacterial culture and speciation, as previously described [15]. Tissues included retropharyngeal, parotid and submandibular lymph nodes, tonsils and lung samples. 

Ethical approval for opportunistic sample collection was granted by Stellenbosch University Animal Care and Use Committee (ACU-2019-9081 and ACU-2019-9086). Permission to perform animal research in terms of section 20 of the Animal Diseases Act was granted by the South African Department of Agriculture, Land Reform and Rural Development (DALRRD), formerly the Department of Agriculture, Forestry and Fisheries (DAFF), South Africa (12/11/1/7/2). All buffaloes were handled by private wildlife veterinarians and game capture teams according to their guidelines. ARRIVE guidelines for reporting animal research have been followed as much as possible (https://arriveguidelines.org/). 

### 2.2. Characterisation of NTMs from Mycobacterial Cultures 

Saline oronasal swab samples, BALF and tissue samples underwent mycobacterial culture in the biosafety level 3 (BSL-3) laboratory at Stellenbosch University, as previously described [15], using a 500 µL aliquot of each oronasal swab media or lavage fluid, and approximately 1 cm^3^ of tissue. Samples were cultured in the BACTEC^TM^ MGIT^TM^ 960 TB System (BD) for at least 56 days, after which a sample with no growth was regarded as a culture negative result, and no downstream analysis performed. Aliquots of culture-positive samples were boiled for 30 min at 99 °C before removal from the BSL-3 facility and downstream testing (Figure 3). 

To identify the mycobacterial species present, PCR reactions were performed on boiled mycobacterial cultures in a Veriti^TM^ 96-Well Thermal Cycler (Applied Biosystems, Waltham, MA, USA) using previously described primer set sequences (Table 1), namely *rpoB* [16] and *hsp*65 [17], followed by Sanger sequencing of amplicons. To establish the presence of virulent factor genes *esat-6* and *cfp-10* in the mycobacterial cultures, previously described *esat*-6 and *cfp*-10 primer sets were used to perform these PCRs (Figure 3), but not for NTM species identification [18,19]. A reaction volume of 25 µL per PCR amplification contained 12.5 µL OneTaq Hot Start 2x master mix (New England Biolabs, Ipswich, MA, USA), 0.8 µM of each appropriate primer pair, 5.5 µL sterile water, and 3 µL aliquots of boiled culture media. No-template controls were included, and *M. bovis* DNA was used as a positive control. Cycling conditions for the *rpoB* PCR assay consisted of 1 cycle at 95 °C for 1 min, followed by 35 cycles at 94 °C for 30 s, 64 °C for 30 s and 72 °C for 60 s, and a final elongation step of 5 min at 72 °C. The other target primer pairs (*hsp*65, *esat*-6 and *cfp*-10) were used in the PCRs with the following cycling conditions: 1 cycle initial denaturation at 95 °C for 15 min, followed by 40 cycles of denaturation (94 °C for 30 s), annealing for 1 min (Table 1) and elongation (72 °C for 30 s). Final elongation took place at 72 °C for 10 min. Presence of the amplified products was confirmed by 1% agarose gel electrophoresis, followed by gel imaging using the ChemiDoc M.D. Universal Hood III Gel Documentation System (Bio-Rad Laboratories, Hercules, CA, USA). Amplicons were sent to the Central Analytical Facility (CAF), Stellenbosch University, for Sanger sequencing. 

Sequence pairwise alignments were performed using the BioEdit Sequence Alignment Editor (Version 7.2.5, Tom Hall, Vista, CA, USA). Sequence contigs were analysed with the NCBI nucleotide Basic Local Alignment Search Tool (BLASTn) to find sequence matches in the NCBI database. Species identity was assigned as follows: the threshold for percent identity of the *hsp*65 or *rpoB* Sanger sequence was set at ≥90% similarity to the BLAST reference sequence; if the species assignment was concordant for *hsp*65 and *rpoB* gene sequences, and the similarity was ≥90% for both, the species identification was recorded. If the gene sequences did not result in an agreed species identity, but similarity was ≥90% for that gene sequence match, species identification was recorded. If the desired sequence similarity was not achieved for either of the genes (i.e., <90% similarity), the isolates were regarded as unidentified at species level, and defined as “unidentifiable mycobacteria”. If more than one species was identified per isolate, using the criteria above, the top five species identified were selected for the particular isolate. Grouping into NTM complexes was performed according to the classification by Fedrizzi et al. [3].

Mycobacterial cultures in which MTBC members were detected with *hsp*65 or *rpoB* PCR underwent an additional PCR to confirm these findings. The selected PCR targeted genetic region of difference (RD) 1, 4, 9 and 12, and primers were used as previously described [20]. A 25 µL reaction contained 12.5 µL OneTaq Hot Start 2× master mix (New England Biolabs), 0.5 µL of each primer, 5.5 µL sterile water, and 1 µL aliquots of boiled culture media. Cycling conditions and electrophoresis gel band analysis were performed as described by Warren et al. [20]. 

### 2.3. Characterisation of NTMs Directly from Oronasal Swabs

The Xpert MTB/RIF Ultra qPCR assay (Ultra, Cepheid, Sunnyvale, CA, USA) was performed to detect MTBC DNA in oronasal swab samples (Figure 3). This qPCR was executed according to the manufacturer’s instructions, with minor modifications [21,22]. The PS-MTM tubes containing swabs were thoroughly vortexed before 700 µL aliquots of each sample and 1300 µL Ultra sample reagent were mixed. Following a 10 s vortex step, samples were incubated for 10 min at room temperature. After a further 5 s vortex, samples were incubated for 5 min, before transferring the entire volume into the sample chamber of the Ultra cartridge for automated processing. Result outputs were automatically classified as “MTB not detected”, “MTB trace detected” or MTB detected “very low”, “low”, “medium”, or “high”. A negative result was regarded as “MTB not detected”. All other outputs were considered a positive Ultra result. Result outputs also included a rifampicin resistance result, but this was irrelevant in the context of this study. 

The line-probe assay, GenoType CM*direct* VER 1.0 (Bruker, Billerica, MA, USA), was used for the detection of mycobacterial species (Figure 3). This assay included modified DNA extraction, multiplex PCR, and reverse hybridisation. The DNA extraction step recommended by the manufacturer could not be used, since the PS-MTM inhibited the multiplex PCR. Therefore, the DNeasy Blood and Tissue kit (Qiagen, Hilden, Germany) was used, according to the manufacturer’s instructions, with slight modifications, to remove PS-MTM during the DNA extraction process. Briefly, 500 µL aliquots of PS-NTM swab samples were centrifuged at room temperature for 15 min at 12,000× *g*. Following removal of the supernatant, the pellet was resuspended in 180 µL phosphate buffered saline (PBS); 25 µL lysozyme (10 mg/mL; Merck, Kenilworth, NJ, USA) was added, after which samples were incubated overnight at 37 °C. Hereafter, the extraction process was performed as described by the manufacturer. The DNA was eluted in a 50 µL volume of buffer. Modified internal controls (IC) were prepared and added to each sample after DNA extraction. Elution buffer (Qiagen (Hilden, Germany)) and provided IC were combined in a 1:1 ratio, incubated at 95 °C for 5 min; then 1 µL aliquots were added to each 50 µL extracted DNA sample. Positive and negative controls were also prepared with IC for PCR. Positive controls were prepared by combining 50 µL extracted *M. bovis* DNA (5 ng/µL), 50 µL elution buffer (Qiagen) and 1 µL IC. Following a 5 min incubation at 95 °C, 50 µL elution buffer was added. Negative controls were prepared by combining 50 µL elution buffer and 1 µL IC. After 5 min incubation at 95 °C, 50 µL elution buffer was added. 

Multiplex PCR assays were performed according to the manufacturer’s instructions, using the GenoType CM*direct* VER 1.0 kit (Bruker). All reagents and biotinylated primers required for amplification were included as proprietary amplification mixes. A MiniAmp^TM^ Thermal Cycler (Thermo Fisher Scientific, Waltham, MA, USA) was used at a ramp rate of ≤2.2 °C/s. 

Reverse hybridisation was performed in the automated GT-Blot 48 hybridisation washer (Bruker), according to the manufacturer’s instructions, using the GenoType CM*direct* VER 1.0 kit (Bruker). After completion of the automated hybridisation, membrane strips were interpreted using the template provided, with a positive result (mycobacteria present) associated with readable bands on the test strip. 

### 2.4. Data Analysis

In this descriptive study, positive and negative mycobacterial culture and clinical sample results are reported as proportions of the total number of buffaloes tested. All mycobacterial culture, PCR, Xpert MTB/RIF Ultra and CM*direct* results are reported in Supplementary Materials. 

## 3. Results

**Figure 4:**
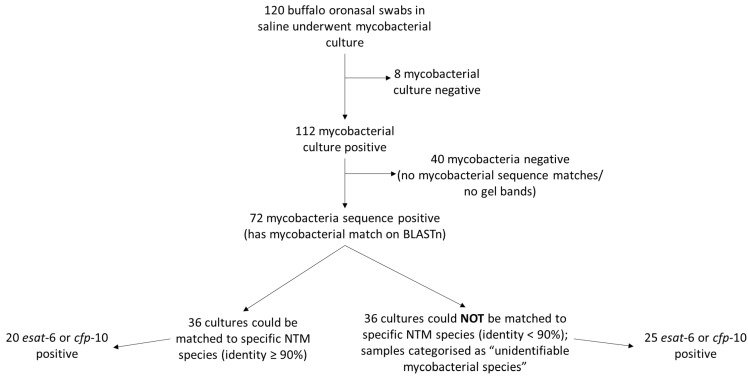
Flow diagram of mycobacterial culture results from buffalo oronasal swabs and Sanger sequencing results from PCRs targeting regions hsp65 and rpoB.

Of the 120 mycobacterial cultures from oronasal swabs, 112 samples grew mycobacteria in MGIT which were identified to the genus level, based on PCR amplicon sequences targeting genomic regions *rpoB* and *hsp*65. Seventy-two of the 112 MGIT positive cultures had a mycobacterial sequence match on NCBI BLASTn (Figure 4). Good quality mycobacterial sequences were obtained from both gene targets (i.e., *hsp*65 and *rpoB*) for only 29 of 72 cultures; 43 isolates had a good quality sequence for only one of the two gene targets. Half of the 72 samples that had a mycobacterial sequence match (*n* = 36) had sequence identity < 90% and were therefore classified as unidentifiable mycobacterial species (i.e., *Mycobacteria* spp.). The remaining 36 buffalo swab cultures (with sequence match identity ≥90%) contained 55 different NTM species and 2 MTBC members (Figure 4; Tables S1 and S2). Multiple NTM species sequence matches that met the identity criteria were found in all but two of these 36 cultures (Table S2). When multiple NTMs were identified in a culture sample, the species were ordered by sequence identity (from highest to smallest) and the top five matches were selected as the mycobacterial species present in the sample. In cases where the percent identity match for the different mycobacterial species were equal, these were included in the group of selected NTM species for that sample (Table S2). *Mycobacterium tuberculosis* and *M. orygis* were detected in two and one buffalo swab cultures, respectively, using the *rpoB* and *hsp*65 amplicon sequences (Tables S1 and S2). However, subsequent genetic analysis using the RD PCR did not confirm these organisms as MTBC members (data not shown). The relative abundance of NTM species cultured from cohort 1 buffaloes was determined based on the number of samples with the identified species; the most abundant NTM species were *M. avium* complex (i.e., *M. avium, M. colombiense, M. intracellulare;* total 49 sequence matches), *M. komanii* (11 sequence matches), *M. novocastrense* (9 sequence matches)*, M. bouchedurhonense* (8 sequence matches) and *M. flavescens* (8 sequence matches). Other NTM species were only found in single culture samples and included *M. ulcerans, M. pyrenivorans, M. paragordonae, M. shottsii* and *M. celeriflavum* (Table S1). 

To determine whether identified NTM species might express cross-reactive antigens with MTBC members, PCRs were performed to detect *esat*-6 or *cfp*-10 genes. Twenty of 36 (56%) NTM-positive buffalo swab cultures were *esat*-6 or *cfp*-10 positive, and 25/36 (69%) buffalo samples that had unidentifiable mycobacteria present were *esat*-6 or *cfp*-10 positive (Figure 4; Table S3). Species of NTM present in samples that were *esat-*6 or *cfp*-10 positive included *M. flavescens, M. komanii, M. avium* complex, *M. smegmatis, M. ulcerans, M. fortuitum, M. abscessus, M. vulneris, M. arosiense, M. diernhoferi, M. gordonae, M. kansasii* and *M. mantenii* (Tables S1 and S2).

The Ultra assay was performed directly on the PS-MTM stored paired buffalo oronasal swabs to determine if samples containing NTMs would lead to false positive Ultra results. Although 106/120 (88%) samples were negative, trace MTBC DNA was detected in 14/120 (12%) swabs. Four trace MTB results were from buffalo swabs that did not contain detectable mycobacteria in culture (Table S3). Five other trace MTB results were reported for PS-MTM oronasal swabs from buffaloes that had cultured mycobacterial species, including *M. fortuitum, M. intracellulare, M. flavescens, M. komanii, M. kansasii* and *M. szulgai*. The remaining five MTB trace results for swabs were from animals with unidentifiable mycobacteria present in their swab culture samples (Table S3). 

The GenoType CMdirect assay was performed with extracted DNA from PS-MTM oronasal swabs to directly speciate NTMs present. Of the 120 buffalo PS-MTM swab samples, 11 were negative, and 3 had an invalid result. Seventy-five of 120 samples had an NTM species match, but the CM*direct* assay was unable to differentiate between mycobacterial species in the remaining 31 samples, which were classified as positive for *Mycobacterium* species (Table S3). Multiple NTM species were detected in some of the samples. Thirteen of 14 buffaloes that had trace MTB Ultra results from swabs were CM*direct* positive for mycobacterial species. Nontuberculous mycobacterial species identified by the CM*direct* assay included *M. fortuitum* complex, *M. szulgai, M. interjectum, M. kansasii* and *M. malmoense.* When mycobacterial species identities from the CM*direct* assay were compared to those identified in cultures using *hsp*65 and *rpoB* PCRs, there were differences for most buffaloes. The CM*direct* assay identified *M. fortuitum* complex in 52 extracted DNA samples from oronasal swabs, although only 2 culture samples were *M. fortuitum* complex positive, based on *hsp*65 and *rpoB* PCR sequencing. Additionally, *M. interjectum* was identified in 24 samples by the CM*direct* assay, but this species was not identified in any mycobacterial culture samples by PCR sequencing. Only one buffalo had concordant results, with *M. fortuitum* complex identified using both CM*direct* and *hsp*65 or *rpoB* PCR assays. (Tables S1–S3).

Since there were a large number of NTMs identified in the oronasal swabs from buffaloes, a small cohort of buffaloes, which had BALF (*n* = 10) or tissue samples (*n* = 19) available, were processed for mycobacterial culture and PCR to investigate whether the NTMs identified in oronasal swabs were more likely to represent environmental contamination (Figure 3). No visible lesions consistent with mycobacteriosis were observed during post-mortem examinations of these individuals. All BALF and tissue samples were mycobacterial culture positive. Of the 10 BALF samples, 6 (60%) had NTMs present, as determined by *hsp*65 PCR, with 4 of 6 samples *esat*-6 or *cfp*-10 PCR positive. Of the tissue samples, 6 of 19 (32%) cultures had mycobacteria present, with one *esat*-6 and *cfp*-10 positive sample. Two of the 6 positive tissue cultures had unidentifiable mycobacteria (identity sequence match < 90%). The most frequent NTM species identified in both BALF and tissue culture samples were *M. avium, M. colombiense* and *M. intracellulare* (Table S4).

## 4. Discussion

In this study, 55 NTM species were identified in oronasal swabs collected from buffaloes originating from historically bTB-free farms in South Africa. Approximately 60% of buffaloes had diverse mycobacterial organisms isolated from culture, and speciated using *hsp*65 and *rpoB* PCR sequencing, with half of these identifiable to the NTM species level. Since exposure to NTMs has been shown to interfere with some immunological tests for bovine TB detection [23], one of the significant findings in this study was that 45 of the 72 cultures that grew *Mycobacteria* spp. from oronasal swabs, contained organisms which contained *esat*-6 or *cfp*-10 genes that encode for immunodominant antigens in *M. bovis.* For rapid mycobacterial identification, which does not involve time-consuming mycobacterial culture, two additional methods were used for direct detection from oronasal swabs. Although none of the buffaloes were suspected to be infected with MTBC, trace MTB results were found for 14 of the 120 oronasal swabs screened using the Xpert MTB/RIF Ultra qPCR assay, suggesting possible cross-reactivity with NTMs or paucibacillary, and possibly nonviable MTBC. The line-probe assay, CM*direct,* was also used for direct characterisation of mycobacteria in oronasal swabs, and 106 of 120 samples were found to contain *Mycobacteria* spp., which indicates a high level of colonisation in buffaloes. The most common NTM species identified were *M. fortuitum* complex and *M. interjectum,* as compared to *M. avium* complex, *M. komanii, M. novocastrense, M. bouchedurhonense* and *M. flavescens* in cultures. The presence of *M. avium* complex was confirmed in culture of post-mortem tissue and BALF samples, which highlights the significance of NTMs in bTB susceptible hosts. 

Accurate identification and characterisation of NTM species in buffaloes are essential for improving bTB diagnostic accuracy and investigating their influence on clinical evaluations and regulatory programs. Opportunistic infections which result in disease, and recognition that NTMs can confound bTB diagnosis, have led to the development of new methods to characterise these species. The use of a combination of PCR primers that target different genetic regions have been shown to improve the discriminatory power of mycobacterial identification [24,25]. In this study we used PCR and Sanger sequencing that targeted two genetic regions, i.e., *rpoB* and *hsp*65. The *rpoB* gene is responsible for encoding the β subunit of RNA polymerase, and primers targeting this region have successfully differentiated mycobacterial species with greater discriminatory power than 16S rRNA sequencing [16,25,26,27,28]. The evolutionarily conserved *hsp65* gene is present in all mycobacterial genomes, but contains unique regions, which allows accurate differentiation of mycobacterial species [17,25,29,30]. 

A large proportion of the 120 oronasal swabs were culture-positive (93%), which could indicate high incidence of environmental mycobacterial contamination in the buffaloes. Since the study used oronasal swabs, which captures respiratory secretions, identified NTMs may not necessarily have infected the buffaloes. However, it has been shown in humans that *M. tuberculosis* bacilli accumulate on the oral epithelia of pulmonary TB patients [31]. Collection of these organisms using swabs provides a less invasive, less time-consuming and less hazardous method for sample collection compared to sputum collection for TB diagnosis [31,32,33]. Likewise, oronasal swabs collected from buffaloes can be used for bTB diagnosis, which are more easily obtainable samples than BALF samples [14]. Used in combination with PS-MTM, which inactivates all pathogens while stabilising nucleic acids, oronasal swab collection may be a safe, rapid, easy and inexpensive method for ante-mortem TB testing of buffaloes [14]. To determine if NTMs were infecting buffaloes, tissue or BALF samples were collected from a small cohort of buffaloes. Sequencing of amplicons from *hsp*65, *esat*-6 and *cfp*-10 PCRs revealed that cultures contained NTMs with the predominant species identified as *M. avium* complex. 

In this study, 55 NTM species were identified in oronasal swab cultures using sequences from hsp65 and *rpoB* PCRs, in addition to a large proportion of buffalo samples having *Mycobacteria* spp. present (60% in total). This finding is consistent with reports which detected NTMs in 64% of cattle in Ghana [34], 7.1% of livestock in the Serengeti region [35], and 16% of livestock in Zambia [2]. Multiple NTM species were also identified in the buffalo oronasal swab cultures, indicating possible mixed infection. This may be expected given the nature of both the sample (oronasal swab) and the NTMs, which are ubiquitous in the environment. The large number of mycobacterial sequence matches could also be related to the sequencing method used, i.e., Sanger sequencing, which, compared to next generation sequencing (NGS), has a shallow depth of coverage. This means that this technique is less capable of differentiating between some bacterial species [36,37]. However, Sanger sequencing is more cost effective than NGS for NTM speciation. 

The predominant NTM species that were identified in mycobacterial cultures included species from the *M. avium* complex, i.e., *M. avium, M. colombiense* and *M. intracellulare,* as well as *M. komanii, M. bouchedurhonense, M. flavescens* and *M. novocastrense.* Other studies also reported the *M. avium* complex and *M. bouchedurhonense* as common pathogenic NTM species that may cause opportunistic disease in a range of animals [2,23,38]. Less frequently detected in this study, but also reported as common potential pathogens in animals are *M. timonense, M. marseillense, M. kansasii, M. fortuitum* and *M. ulcerans* [23,34]*. Mycobacterium avium* complex organisms, especially *M. avium* and *M. intracellulare,* are some of the most common NTM species found in humans [2,39], and cattle [2,34,35]. Exposure to *M. avium* has frequently been associated with tuberculin skin test reactor cattle, and has been found in animals showing TB-like lesions [38]. It has also been reported in a range of other livestock species and wildlife, including deer, bison, wild boar, chickens and buffaloes [39,40,41], but surprisingly not in humans or animals in the Serengeti region of Africa [42]. Other NTM species identified in buffaloes that have been associated with disease in livestock and wildlife include *M. szulgai, M. fortuitum, M. goodii* and *M. abscessus* [23]*. Mycobacterium flavescens, M. elephantis, M. virginiense* and *M. novocastrense* have previously been reported to be associated with TB-like lesions in cattle [34]. Two novel species, i.e., *M. malmesburyense* and *M. komanii,* described by Gcebe et al. [43] in cattle, were identified in the buffalo swab cultures. These species have been shown to contain orthologues for the genes encoding ESAT-6 and CFP-10 antigens. Previous reports of *M. abscessus, M. avium, M. flavescens, M. fortuitum, M. goodii, M. gordonae, M. lentiflavum, M. novocastrense and M. szulgai* have been identified in buffaloes in southern Africa [39,41,42,44]. Due to the wide diversity of NTMs recorded in buffaloes and other hosts, further characterisation of these species is warranted to interrogate their impact on immune responses and pathogenesis. Cross-reactivity in bTB tests has been described in animals exposed to NTMs; *M. fortuitum* and *M. kansasii* may sensitise cattle and lead to false positive results in immunological assays, as well as cause disease in a variety of hosts [7,34,38,45]. Other NTMs, such as *M. flavescens* and *M. szulgai,* contain the RD1 genetic region, which may cause cross-reactivity in bTB diagnostic tests, as well as cause granulomatous disease [18,46,47]. 

Mycobacterial antigens ESAT-6 and CFP-10, which are important for stimulating T-cell responses, are encoded by genes situated in the RD1 genetic region [18]. Although these genes are absent in many NTM species, some NTMs contain these genes, which complicate interpretation of anti-mycobacterial immune responses [1,6,18]. In this study, the majority of cultures of buffalo oronasal swabs were positive for *esat*-6 or *cfp*-10 and included NTM species previously described as containing these regions, i.e., *M. flavescens, M. gordonae, M. kansasii, M. smegmatis, M. komanii, M. vulneris* [6,18,19,43,48].

Since processing for mycobacterial culture can influence which mycobacterial species are identified downstream, direct detection was performed on the PS-MTM preserved oronasal swabs. The CM*direct* assay used DNA that was directly extracted from oronasal swabs and was expected to be paucibacillary. A small proportion (9.2%) of samples were negative on this assay, which was similar to the low number of mycobacteria negative oronasal culture and *hsp*65 and *rpoB* PCR results. Multiple NTM species were found in some samples, similar to what was observed with the oronasal culture samples. Although NTMs were identified in 63% of the samples, the species identified by the CM*direct* assay differed significantly from those found in corresponding culture samples for the majority of buffaloes. Other studies have also described this scenario of misidentification, which appears to be caused by cross-reactivity of the probes in the CM*direct* assay [7,34,49]. In 26% of samples, the CM*direct* assay was unable to speciate the mycobacteria present, which may be because the assay is designed to detect MTBC members and differentiate approximately 20 NTM species that are of clinical importance in humans. Therefore, NTM species of veterinary importance may not be included in this panel, which has also been found in other studies [7,50].

Another technique for direct detection of mycobacteria, the Ultra assay, was performed on the buffalo oronasal swab samples. Since these buffaloes originated from a bTB free herd, negative Ultra results were expected, as the assay is designed to only detect MTBC DNA. Previous studies have shown that the Ultra assay is able to detect MTBC DNA in paucibacillary swabs collected from buffaloes and stored in PS-MTM [14]. The majority of the oronasal swab samples (88%) from buffaloes in the current study were negative in the Ultra assay, with the remaining 12% having trace MTBC DNA detected, which could be false positive results. The Ultra trace category identifies samples with the lowest limit of DNA detection that are *IS*6110 and *IS*1081 positive, but *rpoB* negative [51,52]. Other studies have also found that patient samples with a MTB trace result were smear-negative [51]. Interestingly, 5 of 14 samples with a Ultra trace result had a matching NTM positive culture, determined by *hsp*65 or *rpoB*, and an additional 5 samples had a matching culture with unidentifiable mycobacteria present. Three of the NTM species identified in these culture samples were RD1-containing species (*M. szulgai, M. kansasii* and *M. flavescens*). However, studies report that Ultra has a 100% specificity, and does not detect NTM DNA [53,54]. The high percentage of Ultra negative results in this study further supports the high specificity of this assay, especially since oronasal swabs were used, which contain a large number of environmental organisms. An assay that can discriminate between MTBC and NTM is essential to identify and investigate species that may influence bTB diagnostic test interpretation. 

Limitations of the study included the use of Sanger sequencing rather than NGS, which would have provided deeper coverage to identify mycobacteria present in the cultures and use of a commercial line-probe assay that is designed for human diagnostics, rather than for veterinary use. Furthermore, samples were opportunistically collected, so paired tissue and oronasal swabs from individual animals were unavailable, nor were oronasal samples from buffalo in bTB endemic regions. Oronasal swabs are also not the optimal sample type to use when investigating NTM infection but provide an easily accessible sample. Good quality mycobacterial sequences for both *rpoB* and *hsp*65 amplicons were only obtained for 40% of the mycobacterial cultures, although the majority of cultures had good quality sequences for one of the genes. The inability to generate a sequence for both genes has also been reported in other studies [38]. This may have been caused by the primer not efficiently interacting with the template due to differences between the primer and targeted NTM sequences. The quality of DNA in boiled cultures may have influenced sequencing with the specific primer as well as the formation of secondary structures during PCR [38]. The lower sequence identity value (<90%) in some culture samples may have been caused by a suboptimal sequence amplified during PCR or Sanger sequencing procedures, or the sequences may have a lower similarity match since there were novel NTM variants that have not been previously reported [38]. 

## 5. Conclusions

This study has shown that African buffaloes harbour abundant, diverse NTM species in oronasal secretions, BALF, and tissues. A number of studies have reported the same NTM species in different animal hosts and environmental samples in a study area, indicating that NTMs are ubiquitous and have the potential to colonise or opportunistically infect different hosts. In South Africa, since African buffaloes are important maintenance hosts of bTB, and are required to undergo diagnostic testing prior to movement. However, exposure to NTMs can confound bTB test interpretation. Therefore, studies filling knowledge gaps regarding NTM characterisation, occurrence, and influence on host immune responses in bTB susceptible hosts are essential to improve mycobacterial disease management strategies. 

## Figures and Tables

**Table 1:** Oligonucleotide sequences and PCR conditions for mycobacterial speciation of oronasal swab cultures from African buffaloes.

Primer Name	Forward Primer Sequence (5′–3′)	Reverse Primer Sequence (5′–3′)	Annealing Temperature	Product Size
*rpoB*	GGCAAGGTCACCCCGAAGGG	AGCGGCTGCTGGGTGATCATC	64 °C	±764 bp
*hsp65*	ACCAACGATGGTGTGTCCAT	CTTGTCGAACCGCATACCCT	60 °C	±439 bp
*esat-6*	CATGACAGAGCAGCAGTG	GCCCTATGCGAACATCCC	60 °C	±292 bp
*cfp-10*	GTAGCCCGGGATGGCAGAGATGAAGACCGATGCC	TCAGAAGCCCATTTGCGAGGACAGC	60 °C	±300 bp
bp; base pairs				

## Data Availability

Correspondence and requests for materials should be addressed to W.J.G.

## Supplementary Materials

The following supporting information can be downloaded at: https://www.mdpi.com/article/10.3390/microorganisms10091861/s1, Table S1: Number of sequence matches for each mycobacterial species identified in African buffalo oronasal swab cultures using *hsp*65 and *rpoB* PCR and Sanger sequencing. Table S2: Mycobacterial species matches (sequence identity ≥90%) in African buffalo oronasal swab cultures, as determined by *hsp*65 and *rpo*B PCRs and Sanger sequencing. Presence or absence of *esat*-6 or *cfp*-10 in the cultures are indicated. Table S3: Test results of Ultra and CM*direct* assays performed on oronasal swab samples stored in PrimeStore^®^ MTM. The table also shows the results for mycobacterial culture from oronasal swabs, and PCR and Sanger sequencing with *hsp*65, *rpoB*, *esat*-6 and *cfp*-10 primers performed on these samples from 120 buffaloes. Table S4: Mycobacterial sequence matches in buffalo tissue (*n* = 6) and bronchoalveolar lavage fluid (*n* = 6) cultures using *hsp*65 PCR and Sanger sequencing. Samples with BLASTn sequence matches <90% were defined as having an unidentifiable mycobacterial species.
