# Supplementary material for: Identification and Characterisation of Nontuberculous Mycobacteria in African Buffaloes (Syncerus caffer), South Africa
Source: Microorganisms. 2022 Sep 17;10(9):1861. doi: 10.3390/microorganisms10091861 (PMC9503067; doi:10.3390/microorganisms10091861)
Supplement: Supplementary file 1 [file microorganisms-10-01861-s001.zip › microorganisms-1909542-supplementary.pdf]

Table S1: Number of sequence matches for each mycobacterial species identified in African buffalo oronasal swab cultures using *hsp* 65 and *rpoB* PCR and Sanger sequencing.

Species	Total # of matches
M. avium	19
M. intracellulare	16
M. colombiense	14
M. komanii	11
M. novocastrense	9
M. bouchedurhonense	8
M. flavescens	8
M. lehmannii	6
M. rutilum	6
M. kansasii	3
M. neumannii	3
M. phlei	3
M. psychrotolerans	3
M. vulneris	3
M. arosiense	2
M. fortuitum	2
M. kyorinense	2
M. lacus	2
M. lentiflavum	2
M. mantenii	2
M. marseillense	2
M. timonense	2
M. tuberculosis*	2
M. abscessus	1
M. arupense	1
M. asiaticum	1
M. branderi	1
M. celeriflavum	1
M. chlorophenolicum	1
M. chubuense	1
M. conceptionense	1
M. crocinum	1
M. diernhoferi	1
M. elephantis	1
M. fukienense	1
M. goodii	1
M. gordonae	1
M. hackensackense	1
M. kubicae	1
M. lepromatosis	1
M. liflandii	1
M. malmesburyense	1
M. massiliense	1
M. orygis*	1
M. paragordonae	1
M. paraterrae	1
M. parmense	1

M. pyrenivorans	1
M. rufum	1
M. saopaulense	1
M. shottsii	1
M. smegmatis	1
M. szulgai	1
M. tusciae	1
M. ulcerans	1
M. vicinigordonae	1
M. virginiense	1

*** MTBC members were identified by *hsp* 65 or *rpoB* amplification, but RD PCR (Warren et al 2001) showed that mycobacteria in the cultures were not MTBC members

Table S2: Mycobacterial species matches (sequence identity ≥ 90%) in African buffalo oronasal swab cultures, as determined by *hsp* 65 and *rpoB* PCRs and Sanger sequencing. Presence or absence of *esat-* 6 or *cfp* -10 in the cultures are indicated.

Animal ID	Mycobacterial species	*esat-* 6/ *cfp* -10
S24	*M. flavescens; M. komanii; M. novocastrense; M. phlei; M. rutilum*	Positive
S23	*M. avium; M. colombiense; M. intracellulare*	Negative
S26	*M. avium; M. colombiense; M. intracellulare*	Positive
S18	*M. flavescens; M. komanii; M. novocastrense; M. phlei; M. rutilum*	Positive
S21	*M. avium; M. colombiense; M. intracellulare*	Positive
S22	*M. arosiense; M. avium; M. bouchedurhonense; M. colombiense; M. mantenii*	Negative
S31	*M. avium; M. colombiense*	Positive
S47	*M. avium; M. crocinum; M. fortuitum; M. goodii; M. intracellulare; M. lentiflavum; M. lepromatosis; M. liflandii; M. orygis*; M. paraterrae; M. rufum; M. shottsii; M. smegmatis; M. tuberculosis*; M. ulcerans; M. virginiense*	Positive
S55	*M. avium; M. intracellulare; M. kansasii; M. kyorinense; M. lacus*	Negative
S3	*M. flavescens; M. komanii; M. lehmannii; M. novocastrense; M. psychrotolerans*	Negative
S8	*M. avium; M. bouchedurhonense; M. colombiense; M. intracellulare; M. vulneris*	Negative
S10	*M. avium; M. colombiense; M. intracellulare; M. kubicae; M. parmense*	Negative
S11	*M. avium; M. bouchedurhonense; M. colombiense*	Negative
S38	*M. komanii; M. lehmannii; M. novocastrense; M. pyrenivorans; M. rutilum*	Positive
S39	*M. abscessus; M. conceptionense; M. fukienense; M. massiliense; M. saopaulense*	Positive
S40	*M. komanii; M. lehmannii; M. novocastrense; M. rutilum; M. tusciae*	Negative
S79	*M. flavescens; M. komanii; M. malmesburyense; M. novocastrense; M. phlei*	Negative
S80	*M. avium; M. bouchedurhonense; M. colombiense; M. intracellulare; M. vulneris*	Positive
S84	*M. fortuitum*	Negative
S83	*M. arupense; M. avium; M. celeriflavum; M. colombiense; M. komanii*	Positive
S86	*M. arosiense; M. avium; M. bouchedurhonense; M. colombiense; M. mantenii*	Positive
S61	*M. diernhoferi; M. hackensackense*	Positive
S64	*M. avium; M. intracellulare; M. marseillense*	Negative
S65	*M. avium; M. bouchedurhonense; M. colombiense; M. intracellulare*	Negative
S69	*M. komanii; M. psychrotolerans; M. rutilum*	Negative
S118	*M. avium; M. bouchedurhonense; M. colombiense; M. intracellulare*	Positive
S94	*M. elephantis; M. flavescens; M. lehmannii; M. neumannii; M. novocastrense*	Positive
S102	*M. intracellulare*	Positive
S103	*M. intracellulare; M. timonense*	Negative
S89	*M. asiaticum; M. gordonae; M. lentiflavum; M. paragordonae; M. vicinigordonae*	Positive
S111	*M. avium; M. intracellulare; M. kansasii; M. marseillense; M. timonense*	Positive
S106	*M. chlorophenolicum; M. chubuense; M. flavescens; M. komanii; M. psychrotolerans*	Positive
S101	*M. flavescens; M. komanii; M. lehmannii; M. neumannii; M. novocastrense; M. rutilum*	Negative
S105	*M. flavescens; M. komanii; M. lehmannii; M. neumannii; M. novocastrense*	Positive
S113	*M. avium; M. bouchedurhonense; M. colombiense; M. intracellulare; M. vulneris*	Positive
S115	*M. avium; M. branderi; M. intracellulare; M. kansasii; M. kyorinense; M. lacus; M. szulgai; M. tuberculosis**	Negative

* MTBC members were identified by *hsp* 65 or *rpoB* amplification, but RD PCR (Warren et al 2001) showed that mycobacteria in the cultures were not MTBC members

Table S3: Test results of Ultra and CM*direct* assays performed on oronasal swab samples stored in PrimeStore MTM. The table also shows the results for mycobacterial cultures from saline oronasal swabs, and PCR and Sanger sequencing with *hsp* 65, *rpoB* , *esat* -6 and *cfp* -10 primers performed on these samples from 120 buffaloes.

	**PrimeStore MTM oronasal swab samples**		**Mycobacterial cultures from saline oronasal swab samples**					
**Animal ID**	**Xpert MTB/RIF Ultra**	**Genotype CM*direct***	**Mycobacterial culture (MGIT)**	**NTM present**		**Unidentifiable mycobacterial species**	**RD1 sequence matches**	
				***hsp* 65**	***rpoB***	**% Identity < 90%**	***ESAT-* 6**	***CFP* -10**
S1	MTB not detected	Negative	Positive	N/A	N/A	Yes	Negative	Negative
S2	MTB not detected	*M. fortuitum*	Positive	Negative	Negative		Negative	Negative
S3	MTB not detected	Mycobacterial species	Positive	Positive	Negative		Negative	Negative
S4	MTB not detected	*M. fortuitum*	Positive	Negative	Negative		Negative	Negative
S5	MTB not detected	Negative	Positive	N/A	N/A	Yes	Negative	Positive
S6	MTB not detected	Negative	Positive	Positive	Negative	Yes	Negative	Negative
S7	MTB not detected	Negative	Positive	Negative	Negative		Negative	Negative
S8	MTB not detected	*M. fortuitum*	Positive	Positive	Positive		Negative	Negative
S9	MTB not detected	Negative	Positive	N/A	N/A	Yes	Negative	Positive
S10	MTB not detected	Negative	Positive	Positive	Negative		Negative	Negative
S11	MTB not detected	*M. szulgai*	Positive	Positive	Positive		Negative	Negative
S12	MTB not detected	Mycobacterial species	Positive	Negative	Negative		Negative	Negative
S13	MTB not detected	*M. szulgai*	Positive	N/A	N/A	Yes	Negative	Positive
S14	MTB not detected	*M. fortuitum*	Positive	N/A	N/A	Yes	Negative	Positive
S15	MTB trace detected	*M. fortuitum; M. szulgai*	Positive	N/A	N/A	Yes	Negative	Negative
S16	MTB not detected	*M. fortuitum*	Negative	N/A	N/A		N/A	N/A
S17	MTB not detected	Mycobacterial species	Negative	N/A	N/A		N/A	N/A
S18	MTB not detected	*M. szulgai*	Positive	Positive	Positive		Positive	Positive
S19	MTB not detected	*M. fortuitum*	Negative	N/A	N/A		N/A	N/A
S20	MTB not detected	*M. fortuitum; M. szulgai*	Positive	Negative	Negative		Negative	Positive
S21	MTB not detected	*M. fortuitum*	Positive	Positive	Positive		Positive	Positive
S22	MTB not detected	Mycobacterial species	Positive	Positive	Positive		Negative	Negative
S23	MTB not detected	*Mycobacterial species*	Positive	Positive	Negative		Negative	Negative
S24	MTB not detected	*M. fortuitum*	Positive	Positive	Positive		Negative	Positive
S25	MTB not detected	Invalid	Positive	Negative	Negative		Negative	Positive
S26	MTB not detected	*M. fortuitum*	Positive	Positive	Positive		Negative	Positive

S27	MTB not detected	*M. fortuitum*	Negative	N/A	N/A		N/A	N/A
S28	MTB not detected	*M. fortuitum*	Positive	Negative	Negative		Negative	Negative
S29	MTB not detected	*M. fortuitum*	Positive	Negative	Negative		Negative	Negative
S30	MTB not detected	*M. fortuitum*	Positive	Negative	Negative		Negative	Negative
S31	MTB not detected	Mycobacterial species	Positive	Positive	Negative		Negative	Positive
S32	MTB not detected	*M. fortuitum*	Positive	Negative	Negative		Negative	Positive
S33	MTB not detected	*M. fortuitum; M. intracellulare*	Positive	Negative	Negative		Negative	Negative
S34	MTB not detected	*M. fortuitum*	Positive	N/A	N/A	Yes	Negative	Positive
S35	MTB not detected	Mycobacterial species	Positive	Negative	Negative		Negative	Negative
S36	MTB not detected	*M. fortuitum; M. szulgai*	Positive	Negative	Negative		Negative	Negative
S37	MTB not detected	*M. fortuitum*	Positive	Negative	Negative		Negative	Negative
S38	MTB not detected	Mycobacterial species	Positive	Positive	Negative		Negative	Positive
S39	MTB not detected	Mycobacterial species	Positive	Positive	Negative		Negative	Positive
S40	MTB not detected	Mycobacterial species	Positive	Positive	Negative		Negative	Negative
S41	MTB not detected	*M. interjectum*	Positive	N/A	N/A	Yes	Negative	Negative
S42	MTB not detected	*M. fortuitum*	Positive	N/A	N/A	Yes	Negative	Positive
S43	MTB not detected	*M. fortuitum*	Positive	Negative	Negative		Negative	Positive
S44	MTB not detected	*M. fortuitum; M. malmoense*	Positive	Negative	Negative		Negative	Negative
S45	MTB not detected	*M. szulgai*	Positive	Negative	Negative		Negative	Positive
S46	MTB not detected	*M. fortuitum; M. kansasii*	Positive	N/A	N/A	Yes	Negative	Negative
S47	MTB not detected	*M. fortuitum; M. interjectum*	Positive	Positive	Negative		Negative	Positive
S48	MTB not detected	*M. fortuitum; M. interjectum*	Positive	N/A	N/A	Yes	Negative	Positive
S49	MTB not detected	*M. fortuitum; M. interjectum*	Positive	Negative	Negative		Negative	Positive
S50	MTB not detected	Mycobacterial species	Positive	Negative	Negative		Negative	Positive
S51	MTB not detected	Mycobacterial species	Positive	N/A	N/A	Yes	Negative	Negative
S52	MTB not detected	*M. interjectum*	Positive	Negative	Negative		Negative	Negative
S53	MTB not detected	*M. interjectum*	Positive	N/A	N/A	Yes	Negative	Positive
S54	MTB not detected	Mycobacterial species	Positive	Negative	Negative		Negative	Negative
S55	MTB not detected	*M. fortuitum*	Positive	Positive	Negative		Negative	Negative
S56	MTB not detected	*M. interjectum*	Positive	Negative	Negative		Negative	Negative
S57	MTB not detected	*M. szulgai*	Positive	Negative	Negative		Negative	Negative

S58	MTB not detected	*M. chelonae; M. interjectum*	Positive	Negative	Negative		Negative	Negative
S59	MTB trace detected	*M. fortuitum; M. intracellulare; M. interjectum; M. malmoense*	Positive	N/A	N/A	Yes	Negative	Positive
S60	MTB not detected	*M. fortuitum; M. szulgai*	Positive	Negative	Negative		Negative	Negative
S61	MTB not detected	Mycobacterial species	Positive	Positive	Negative		Negative	Positive
S62	MTB not detected	*Negative*	Positive	N/A	N/A	Yes	Negative	Negative
S63	MTB not detected	*M. fortuitum*	Positive	N/A	N/A	Yes	Negative	Negative
S64	MTB not detected	*M. fortuitum*	Positive	Positive	Negative		Negative	Negative
S65	MTB not detected	*M. fortuitum*	Positive	Positive	Negative		Negative	Negative
S66	MTB not detected	Negative	Positive	Negative	Negative		Negative	Positive
S67	MTB not detected	Mycobacterial species	Positive	Negative	Negative		Negative	Negative
S68	MTB not detected	*M. fortuitum*	Positive	Negative	Negative		Negative	Negative
S69	MTB not detected	Negative	Positive	Positive	Negative		Negative	Negative
S70	MTB trace detected	*M. fortuitum*	Positive	Negative	Negative		Negative	Negative
S71	MTB not detected	Mycobacterial species	Positive	N/A	N/A	Yes	Negative	Positive
S72	MTB trace detected	*M. fortuitum*	Positive	Negative	Negative		Negative	Negative
S73	MTB not detected	Mycobacterial species	Positive	Negative	Negative		Negative	Positive
S74	MTB not detected	*M. fortuitum; M. szulgai*	Positive	N/A	N/A	Yes	Negative	Positive
S75	MTB not detected	Mycobacterial species	Positive	N/A	N/A	Yes	Negative	Positive
S76	MTB not detected	*M. kansasii; M. malmoense*	Negative	N/A	N/A		N/A	N/A
S77	MTB not detected	Negative	Negative	N/A	N/A		N/A	N/A
S78	MTB not detected	Mycobacterial species	Positive	Negative	Negative		Negative	Negative
S79	MTB not detected	*M. abscessus; M. fortuitum*	Positive	Positive	Positive		Negative	Negative
S80	MTB not detected	Mycobacterial species	Positive	Positive	Positive		Negative	Positive
S81	MTB trace detected	*M. fortuitum*	Negative	N/A	N/A		N/A	N/A
S82	MTB not detected	Invalid	Positive	Negative	Negative		Negative	Negative
S83	MTB not detected	*M. fortuitum; M. szulgai*	Positive	Positive	Positive		Positive	Negative
S84	MTB trace detected	Mycobacterial species	Positive	Positive	Negative		Negative	Negative
S85	MTB not detected	Mycobacterial species	Negative	N/A	N/A		N/A	N/A
S86	MTB not detected	*M. kansasii*	Positive	Positive	Positive		Positive	Positive
S87	MTB not detected	*M. fortuitum; M. gordonae*	Positive	Negative	Negative		Negative	Negative

S88	MTB not detected	*M. interjectum; M. malmoense*	Positive	N/A	N/A	Yes	Negative	Positive
S89	MTB not detected	Negative	Positive	Positive	Positive		Negative	Positive
S90	MTB trace detected	Mycobacterial species	Positive	N/A	N/A	Yes	Negative	Negative
S91	MTB trace detected	*M. interjectum*	Positive	N/A	N/A	Yes	Negative	Positive
S92	MTB not detected	*M. fortuitum*	Positive	N/A	N/A	Yes	Negative	Positive
S93	MTB not detected	Mycobacterial species	Positive	N/A	N/A	Yes	Negative	Positive
S94	MTB not detected	*M. interjectum*	Positive	Positive	Positive		Negative	Positive
S95	MTB not detected	*M. fortuitum; M; interjectum*	Positive	N/A	N/A	Yes	Negative	Positive
S96	MTB not detected	Mycobacterial species	Positive	N/A	N/A	Yes	Negative	Positive
S97	MTB trace detected	Mycobacterial species	Positive	N/A	N/A	Yes	Negative	Negative
S98	MTB not detected	M. fortuitum; M. interjectum	Positive	N/A	N/A	Yes	Negative	Positive
S99	MTB trace detected	*M. interjectum*	Positive	Negative	Negative		Negative	Negative
S100	MTB not detected	*M. fortuitum*	Positive	Negative	Negative		Negative	Positive
S101	MTB not detected	*M. interjectum*	Positive	Positive	Negative		Negative	Negative
S102	MTB trace detected	Mycobacterial species	Positive	Positive	Positive		Negative	Positive
S103	MTB trace detected	Invalid	Positive	Positive	Negative		Negative	Negative
S104	MTB not detected	*M. szulgai*	Positive	N/A	N/A	Yes	Negative	Positive
S105	MTB not detected	*M. fortuitum; M. interjectum*	Positive	Positive	Positive		Negative	Positive
S106	MTB trace detected	*M. interjectum*	Positive	Positive	Negative		Negative	Positive
S107	MTB not detected	*M. interjectum*	Positive	Negative	Negative		Negative	Positive
S108	MTB not detected	*M. fortuitum; M. interjectum*	Positive	N/A	N/A	Yes	Negative	Positive
S109	MTB not detected	*M. fortuitum*	Positive	N/A	N/A	Yes	Negative	Positive
S110	MTB not detected	*M. fortuitum; M. interjectum*	Positive	N/A	N/A	Yes	Negative	Positive
S111	MTB not detected	*M. interjectum*	Positive	Positive	Negative		Negative	Positive
S112	MTB not detected	Mycobacterial species	Positive	Negative	Negative		Negative	Negative
S113	MTB not detected	*M. fortuitum; M. interjectum*	Positive	Positive	Positive		Negative	Positive
S114	MTB not detected	*M. fortuitum*	Positive	N/A	N/A	Yes	Negative	Positive
S115	MTB trace detected	Mycobacterial species	Positive	Positive	Negative		Negative	Negative
S116	MTB not detected	Mycobacterial species	Positive	N/A	N/A	Yes	Negative	Positive
S117	MTB not detected	*M. fortuitum; M. szulgai*	Positive	Negative	Negative		Negative	Positive

S118	MTB not detected	*M. szulgai*	Positive	Positive	Positive		Negative	Positive
S119	MTB not detected	Mycobacterial species	Positive	N/A	N/A	Yes	Negative	Negative
S120	MTB not detected	*M. interjectum*	Positive	Negative	Negative		Negative	Negative
Total positive	14	106	112	36		36	4	56

Table S4: Mycobacterial sequence matches in buffalo tissue (n = 6) and bronchoalveolar lavage fluid (n = 6) cultures using *hsp* 65 PCR and Sanger sequencing. Samples with BLASTn sequence matches < 90% were defined as having an unidentifiable mycobacterial species

**Mycobacterial species**	**Tissue culture**						**Bronchoalveolar lavage fluid culture**					
	**TC1**	**TC2**	**TC3**	**TC4**	**TC5**	**TC6**	**LC1**	**LC2**	**LC3**	**LC4**	**LC5**	**LC6**
M. alvei												X
M. avium	X	X	X	X					X	X		
M. bouchedurhonense	X											
M. colombiense	X	X	X	X					X	X		
M. europaeum							X	X				
M. fortuitum											X	X
M. goodii											X	
M. houstonense												X
M. intracellulare	X	X	X	X					X	X		
M. paraense		X										
M. parascrofulaceum							X	X				
M. parmense		X		X								
M. peregrinum											X	
M. piscinum											X	
M. saskatchewanense							X	X				
M. scrofulaceum							X	X				
M. setense												X
M. smegmatis											X	
M. szulgai							X	X				
M. vulneris	X											
M. wolinskyi												X
Unidentifiable mycobacterial species					X	X						

TC 1-6 and LC 1-6 are the sample numbers from tissue cultures and BALF cultures, respectively
